# Effect of Hyperoxia and Hypercapnia on Tissue Oxygen and Perfusion Response in the Normal Liver and Kidney

**DOI:** 10.1371/journal.pone.0040485

**Published:** 2012-07-06

**Authors:** Hai-Ling Margaret Cheng

**Affiliations:** 1 Physiology & Experimental Medicine, The Hospital for Sick Children Research Institute, Toronto, Ontario, Canada; 2 Diagnostic Imaging, The Hospital for Sick Children, Toronto, Ontario, Canada; 3 Department of Medical Biophysics, University of Toronto, Toronto, Ontario, Canada; The University of Chicago, United States of America

## Abstract

**Objective:**

Inhalation of air with altered levels of oxygen and carbon dioxide to manipulate tissue oxygenation and perfusion has both therapeutic and diagnostic value. These physiological responses can be measured non-invasively with magnetic resonance (MR) relaxation times. However, interpreting MR measurements is not straight-forward in extra-cranial organs where gas challenge studies have only begun to emerge. Inconsistent results have been reported on MR, likely because different organs respond differently. The objective of this study was to elucidate organ-specific physiological responses to gas challenge underlying MR measurements by investigating oxygenation and perfusion changes in the normal liver and kidney cortex.

**Materials and Methods:**

Gas challenges (100% O_2_, 10% CO_2_, and carbogen [90% O_2_+10% CO_2_]) interleaved with room air was delivered to rabbits to investigate their effect on tissue oxygenation and perfusion. Real-time fiber-optic measurements of absolute oxygen and relative blood flow were made in the liver and kidney cortex.

**Results:**

Only the liver demonstrated a vasodilatory response to CO_2_. Perfusion changes to other gases were minimal in both organs. Tissue oxygenation measurements showed the liver responding only when CO_2_ was present and the kidney only when O_2_ was present.

**Conclusion:**

This study reveals distinct physiological response mechanisms to gas challenge in the liver and kidney. The detailed characterization of organ-specific responses is critical to improving our understanding and interpretation of MR measurements in various body organs, and will help broaden the application of MR for non-invasive studies of gas challenges.

## Introduction

Breathing elevated levels of O_2_ (hyperoxia) or CO_2_ (hypercapnia) has both therapeutic and diagnostic value. One important application is to enhance tissue oxygen supply for improving the efficacy of radiation therapy of cancer [1] and the treatment of ischemia [2]. The mechanisms of action differ, as hyperoxia directly increases blood oxygen content while hypercapnia induces a vasodilatory response that augments blood delivery of oxygen and nutrients. Another application is to utilize the potent vasodilatory action of CO_2_ to assess the health of small blood vessels through their vasoreactive response in the study of various cerebrovascular pathologies [3].

Whether the end goal is to increase tissue oxygenation or to assess the responsiveness of blood vessels, a non-invasive means to monitor the effects on oxygen content or perfusion at the tissue level is valuable. Magnetic resonance imaging (MRI) provides non-invasive and sensitive detection of tissue oxygenation through the longitudinal (T_1_) and effective transverse (T_2_*) relaxation times [4]–[6]. Changes in T_1_ stem mainly from molecular O_2_ dissolved in blood plasma and in intra- and extracellular fluids [7], [8]. Changes in T_2_* stem from local field perturbations created by paramagnetic deoxyhemoglobin molecules, which provide a useful indication of the ratio of deoxyhemoglobin to oxyhemoglobin (Hb/HbO_2_) [9]. Oxygenation is only one variable, however, as these MRI parameters are influenced by blood volume and other factors; therefore, their proper interpretation is not straight-forward. The first step towards interpreting T_1_ and T_2_* correctly and understanding their association with underlying physiological responses is to characterize how a gas challenge exerts concomitant and perhaps organ-specific changes on oxygenation and blood flow. The physiology of gas inhalation and its association with MRI measurements is well established in the brain [10]. However, our understanding of the relationship between physiology and MRI parameters is relatively poor in most extra-cranial tissues. The emerging literature in MRI gas inhalation studies in body organs [7], [8], [11]–[17] focus primarily on imaging measurements with very little attempt to elucidate the physiological underpinnings. The reported T_1_ and T_2_* responses in these studies are inconsistent at times and can differ significantly from expected changes observed in brain tissue. These inconsistencies suggest organ-specificity in tissue response, but interpreting these MRI responses remains speculative without physiological validation measurements.

In this study, our aim was to characterize the dynamic response of tissue oxygenation and perfusion to hyperoxia and hypercapnia in the normal rabbit liver and kidney. These two organs are ideal for investigating organ-specificity, as they are known to exhibit distinct physiological responses to altered arterial gas levels. For example, the liver is known to increase blood volume in response to hypercapnia [14], [18]. Conversely, the kidney responds to hypercapnia by reducing blood flow [19], [20]. The physiological characterization obtained in this study will enable us to interpret the MRI findings in our previous study [11]. More importantly, it will expand our understanding of MRI measurements in different organs to improve and broaden the application of MRI to study gas challenges.

## Methods

The study was approved by the Hospital for Sick Children Animal Care Committee (Protocol #8661). All procedures were conducted in accordance with the Canadian Council on Animal Care.

### Animal preparation

Experiments were performed on six New Zealand white rabbits (2.8–3.2 kg). Anaesthesia in rabbits was induced with an intramuscular Akmezine injection followed by 5% isoflurane in oxygen (2 L/min) delivered by a face mask, and a catheter was inserted into the ear vein to maintain hydration (4 ml/kg/h 0.9% NaCl). Rabbits were intubated and spontaneously breathing 2 L/min during each gas challenge, with anaesthesia maintained at 1.5% isoflurane. Isoflurane was selected based on its limited effects on hemodynamics [21]. One ear artery was cannulated for blood gas analysis. Heart rate and oxygen saturation were continuously monitored using a veterinary monitor (Mindray PM– 9000Vet).

### Gas challenges

**Figure 1:**
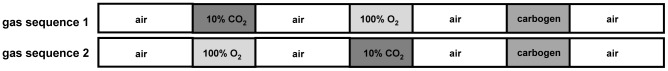
Gas inhalation protocol. Baseline air is delivered in 15 minute blocks. All other gases are delivered in 10 minute blocks.

Figure 1 illustrates the two different gas inhalation sequences applied. Each sequence consisted of cycling through 100% O_2_, 10% CO_2_ (balance air), and carbogen (90% O_2_+10% CO_2_), each delivered in 10 minute blocks. These were interleaved with room air (21% O_2_, balance N_2_) inhalation delivered in 15 minute blocks to allow return to equilibrium. The order of gas delivery was randomized to assess if tissue response was independent of previous gas inhalation history.

### Tissue oxygenation and perfusion measurements

Real-time measurements of tissue oxygenation (pO_2_) and relative perfusion were obtained in the liver and kidney cortex of each animal on the two different gas inhalation protocols. The OxyLite/OxyFlo (Oxford Optronics Ltd., Oxford, UK) fiber-optic system was used to provide minimally invasive (using fiber optic probes 250–450 microns in diameter) and continuous monitoring of tissue perfusion and oxygenation simultaneously. The OxyLite system quantifies absolute tissue pO_2_ levels through fluorescence lifetime decay of a fluorophore at the tip of the probe that is inversely related to pO_2_. These pO_2_ measurements only detect dissolved molecular O_2_ and are insensitive to hemoglobin-bound oxygen. The OxyFlo system uses laser Doppler flowmetry to provide continuous monitoring of blood flow in relative perfusion units (BPU) at a temporal resolution of 2.4 s. The BPU is useful for assessing relative changes in blood flow but should not be used to compare different organs, as different tissues have different optical properties.

To perform invasive measurements, a laparotomy was performed to expose the liver and kidney, and the exposed organs were covered with wet gauze to maintain hydration. One OxyLite channel was used to assess pO_2_ from a probe inserted between liver lobes, and a second channel was used to monitor pO_2_ from a probe inserted approximately 2 mm into the kidney cortex. The two OxyFlo probes were inserted approximately 5 mm away from each of the corresponding oxygen sensors in the liver and kidney. These probe placement strategies were chosen to avoid puncturing the liver and causing bleeding and to ensure kidney measurements were made in the cortex and not in the medulla. To mitigate effects of respiratory motion in the liver, the probes were sutured to the rabbit's stomach so that the probe would follow the path of the liver during the respiratory cycle. Similarly, the probes were taped onto towels wrapped around the kidney to alleviate potential errors due to slight motion. Blood gas samples were collected at the end of each inspired gas challenge and analyzed on a blood gas analyzer (Radiometer ABL 700 Series). The entire procedure, including surgery and real-time monitoring, lasted on average two hours.

### Data analysis

The twelve sets of real-time absolute oxygen and relative blood flow measurements, obtained from all six animals on the two gas sequences, were analyzed in Matlab version 7.8 (MathWorks, Natick, MA). The average oxygenation or perfusion measurement was determined within the time interval of a gas challenge. In the case of an increasing or decreasing response, the maximum or minimum value was determined. These were then averaged over the twelve sets of data to calculate the mean and standard deviation for that particular gas challenge. Analysis of variance (ANOVA) was used to determine differences in oxygenation or perfusion response amongst the various gas challenges, and post-hoc Tukey-Kramer testing was used for multiple comparisons. Significance was declared at a probability value (*P*)<0.05.

## Results

Arterial blood gas analysis results for each inspired gas challenge are provided in Table 1. Carbogen generated similar arterial partial pressure of CO_2_ (paCO_2_) levels to 10% CO_2_ and similar arterial partial pressure of O_2_ (paO_2_) levels to 100% O_2_. Both the heart rate (211–249 min^−1^) and the respiratory rate (24–29 min^−1^) were quite stable throughout the experiment.

**Table 1:** Arterial blood gas measurements.

Gas Challenge	pH	paCO_2_ (mmHg)	paO_2_ (mmHg)	saO_2_ (%)
Baseline air	7.46±0.02	35±3	79±9	94.7±2.3
10% CO_2_	7.20±0.06	70±6	86±8	93.2±2.2
100% O_2_	7.40±0.05	41±5	430±12	99.6±2.5
carbogen	7.18±0.06	71±8	415±29	99.2±2.3

Values are mean ± SD (*N* = 12).

Abbreviations: paCO_2_, arterial partial pressure of CO_2_; paO_2_, arterial partial pressure of O_2_; saO_2_, arterial oxygen saturation.

Gases: air (21%O_2_, balance N_2_), 10% CO_2_ (balance air), carbogen (90% O_2_+10% CO_2_).

**Figure 2:**
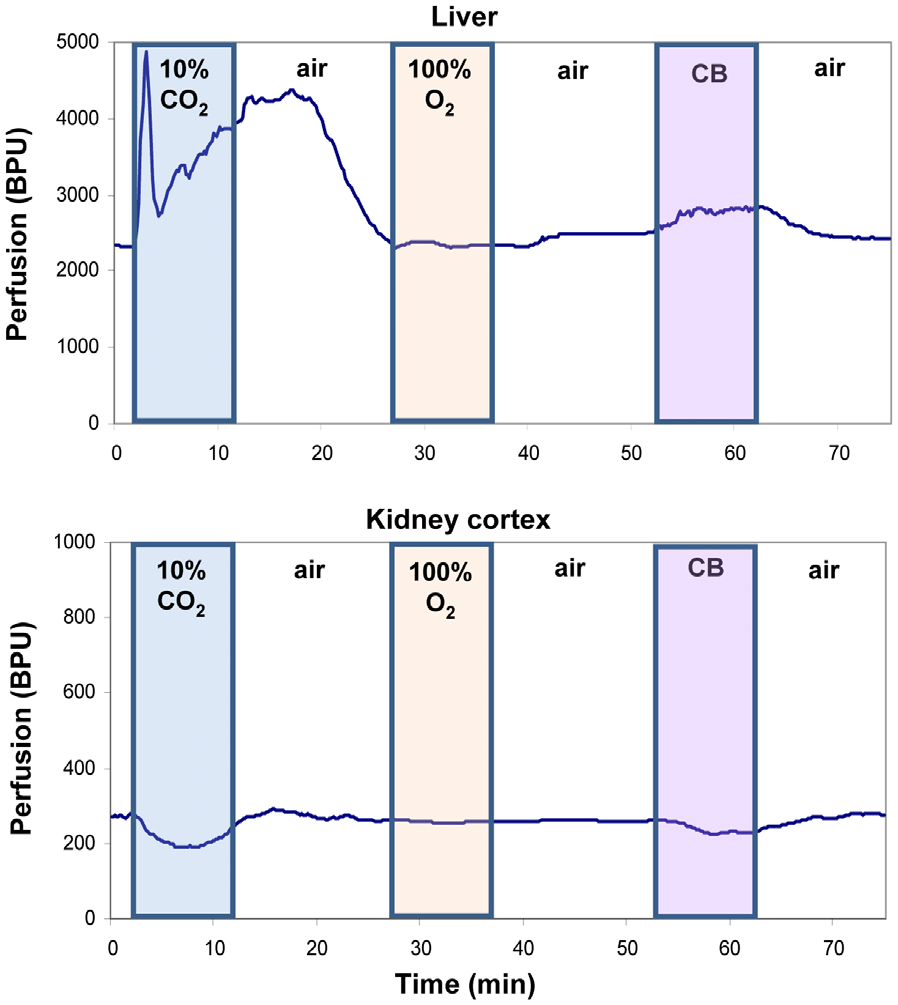
Real-time tissue perfusion response in the liver and kidney cortex. Results are shown for gas sequence 1. CB refers to carbogen. Units are in relative blood perfusion units (BPU).

Figure 2 illustrates the temporal dynamics of perfusion response in both the liver and kidney cortex for gas sequence 1. In the liver, there is a rapid fluctuation in response to CO_2_ that shows a significant overall increase in perfusion. Perfusion returns to baseline upon 100% O_2_ inhalation and is slightly elevated during carbogen breathing. In contrast, perfusion in the kidney decreases slightly in response to CO_2_ but remains relatively stable for the hyperoxic gas mixtures. Similar patterns were observed regardless of the order of gas delivery. Table 2 summarizes these blood flow changes for both gas sequences across all six animals.

**Table 2:** OxyLite/OxyFlo measurements of tissue oxygenation and perfusion.

Gas Challenge	Liver		Kidney	
	pO_2_ (mmHg)	Blood flow (BPU)	pO_2_ (mmHg)	Blood flow (BPU)
Baseline air	25.2±4.5 [19]–[33]	2031±157 [1800–2332]	34.5±4.8 [27–41]	281±30 [242–343]
10% CO_2_	54.2±8.6 * [44–73]	4094±798 * [3002–5201]	38.8±6.4 [28–48]	234±32 * [184–284]
100% O_2_	29.1±4.9 [22]–[36]	2071±209 [1749–2350]	58.8±9.8 * [45–73]	284±30 [247–332]
carbogen	69.2±12.4 * [50–87]	2600±408 * [1834–3144]	73.1±13.0 * [51–91]	256±27 [211–294]

Values are mean ± SD (*N* = 12). Range in square brackets.

Abbreviations: pO_2_, tissue oxygen tension; BPU, blood perfusion units.

Gases: air (21%O_2_, balance N_2_), 10% CO_2_ (balance air), carbogen (90% O_2_+10% CO_2_).

* *P*<0.05 significantly different from baseline (air breathing).

**Figure 3:**
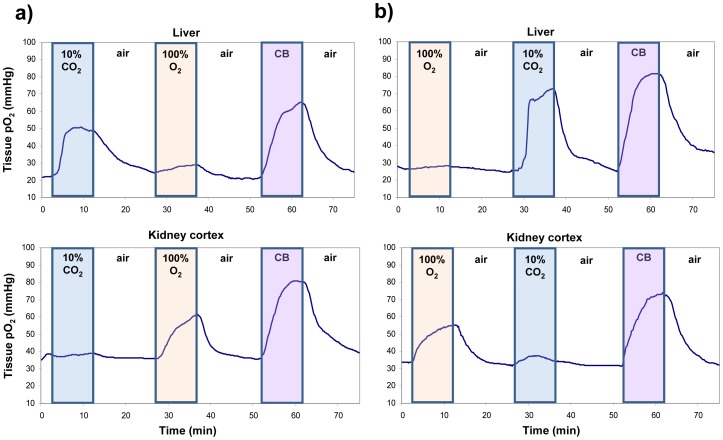
Real-time tissue oxygenation response in the liver and kidney cortex. Results are shown for (a) gas sequence 1 and (b) gas sequence 2. CB refers to carbogen. Units are in absolute mmHg.

Figure 3a illustrates tissue oxygenation response in both the liver and kidney cortex for gas sequence 1. A striking difference was consistently observed between the two organs: minimal changes in oxygenation were obtained on 100% O_2_ in the liver and on 10% CO_2_ in the kidney. In other words, in the liver augmented CO_2_ was necessary to improve oxygenation, whereas in the kidney augmented O_2_ was required. The same behavior was observed for gas sequence 2 (Figure 3b), which suggests that these organ-specific response characteristics are dependent only on the composition but not the history of the gas challenge. A summary of these changes in all animals are given in Table 2.

## Discussion

MRI is a useful non-invasive tool for monitoring the effects of altered inspired O_2_ and CO_2_ levels in the diagnosis and treatment of cancer and microvascular diseases. Hyperoxia increases oxygen delivery through augmenting oxygen dissolved in arterial plasma, which effectively decreases venous Hb/HbO_2_. This is expected to result in a T_1_ decrease/T_2_* increase indicating a higher pO_2_ and lower Hb levels, respectively. Hypercapnia increases oxygen delivery through vasodilation. It is expected to result in a T_1_ increase/T_2_* increase indicating increased blood volume and lower Hb levels, respectively. Unfortunately, these anticipated MRI responses to gas inhalation have not been consistently observed in organs outside of the brain, which raises caution over the interpretation of MRI measurements. For example, the expected T_1_ reduction from hyperoxia has been consistently reported only in vessel-rich tissues, such as spleen and myocardium [7], [8], [13], [16]. Other tissues, such as liver, kidney, and muscle, have shown smaller T_1_ reductions [8], [11], [13] or negligible T_1_ changes [7], [15]. Similarly, the expected T_2_* increase with enhanced oxygen delivery, which is well established in the brain [10], has not been consistently observed in body organs. Lack of T_2_* response in the liver and kidney has been reported in response to pure oxygen [7], [13], [15]. A more recent study [11] investigated a variety of gas mixtures and reported several notable findings, including absent T_2_* changes in the kidney (hypercapnia) and liver (hyperoxia) and a T_2_* decrease in the liver (hypercapnia).

Table 3 provides a unified framework for understanding the association between gas-induced physiological response and MRI relaxation times T_1_ and T_2_*. Using the known responses in the brain and their association with MRI measurements, the distinct tissue pO_2_ and perfusion responses obtained in this study are used to predict T_1_ and T_2_* changes in the liver and kidney. These predicted changes on MRI are then compared to findings from key MRI studies on gas challenge in the liver and kidney. This discussion will hopefully lend better insight into the interpretation of MRI measurements in different tissue types and clarify why different MRI measurements should be expected.

**Table 3:** Physiological changes observed and MRI response to hyperoxia and hypercapnia.

Inspired gas	Organ	pO_2_	BF	Expected changes in MRI relaxation times		Reported changes on MRI in literature (reference number)	
				ΔT_1_	ΔT_2_*	ΔT_1_	ΔT_2_*
hyperoxia	Brain	↑	↓	↓ Increased pO_2_	↑ Decreased venous Hb	↓ 29	↑ 25,26
	Liver	↔	↔	↔ or minimal ↓ Oxygen delivery and tissue pO_2_ response are buffered by portal vein	↔ Hb content is unaltered from stable response in BF and blood oxygen supply	↓ 8,11,13 ↔ 7,15	↔ 7,11,13,15
	Kidney	↑	↔	↓ Increased pO_2_	↑ Decreased venous Hb	↓ 8,11,13,15	↑ 11,12 ↔ 13,15
hypercapnia	Brain	↑	↑	↔ Increased pO_2_ counteracted by increased blood volume	↑ Decreased venous Hb from augmented blood flow	↔ 27	↑ 25,26
	Liver	↑	↑	↑ Increased blood volume	↓ Increased Hb from augmented flow from portal vein relative to hepatic artery	↑ 11	↓ 11,14,17
	Kidney	↔	↔	↔ Stable blood flow response has negligible effect on tissue pO_2_	↔ Hb content is unaltered from stable response in BF and blood oxygen supply	↔ 11	↔ 11

↑ increase; ↓ decrease; ↔ no change.

Abbreviations: pO_2_, tissue oxygen tension; BF, blood flow; Hb, deoxyhemoglobin.

Gases: hyperoxia (100% O_2_), hypercapnia (10% CO_2_, balance air).

Prior to discussing body organs, we will first review brain response to gas inhalation and explain its well established association with T_1_/T_2_*. In the brain, elevated CO_2_ levels (hypercapnia) primarily increases blood flow through vasodilation [22]–[25], which results in a greater venous oxygen saturation. The reduced venous Hb content produces a higher T_2_* [25], [26]. The higher blood flow also improves oxygen delivery to tissue, which should lower T_1_; however, competing effects from a higher blood volume would increase T_1_, thereby resulting in negligible T_1_ changes [27]. Elevated O_2_ levels (hyperoxia) reduces perfusion slightly [23], [25], [28] but increases the arterial oxygen partial pressure (i.e. oxygen dissolved in plasma), which also increases venous oxygen saturation. The results of a higher dissolved oxygen content and lower venous Hb content are, respectively, a lower T_1_ and higher T_2_* [25], [26], [29]. Effects of elevating both O_2_ and CO_2_ (carbogen) are less clear, even though the CO_2_ is added to counteract possible hyperoxia-induced vasoconstriction. The combined effect of carbogen breathing is complex, and variable results have been documented in numerous studies [30]–[32].

In the liver, our study shows that perfusion increases significantly during hypercapnia but remains near baseline during hyperoxia. These observations are consistent with physiological studies showing increased total liver blood flow, mainly through the portal vein, when CO_2_ is elevated [14], [18] and no flow changes when O_2_ is elevated [33], [34]. More illuminating is new physiological evidence of the liver's unique response made through tissue pO_2_ measurements, where we obtained negligible pO_2_ changes on breathing pure oxygen but significant increases when CO_2_ was present (either alone or combined with elevated O_2_). These observations explain many of the reported results in MRI studies. When total blood flow increases during hypercapnia, T_1_ increases to reflect a substantially larger blood volume. Tissue oxygenation also improves from augmented blood flow, but since most of the flow increase stems from the portal vein while hepatic arterial flow reduces to offset flow variations [35], there is an overall increase in Hb/HbO_2_. This effectively lowers the T_2_*, which is opposite to the T_2_* increase seen in the arterial-supplied brain. When pure oxygen was delivered, pO_2_ was unexpectedly stable. The most likely explanation is that since the liver receives mainly portal blood, changes in blood dissolved oxygen are not completely transmitted through the portal circulation to the liver. This response may be a protection mechanism from hyperoxia similar to that found in the retina, although the mode of action is different as retinal vessels constrict. In the liver, the consequence of unaltered blood flow and small increases in blood oxygen in the feeding vessels is a negligible change in T_2_* and slight reduction in T_1_, if any.

In the kidney, perfusion changes were minimal for all gas challenges, although a slight decrease was noted on hypercapnia. These results are consistent with physiological studies showing decreased renal blood flow when CO_2_ is elevated [19], [20] and no changes upon O_2_ elevation [19], [36]. The mechanisms for reduced renal blood flow under hypercapnia are not fully understood, although there is evidence of both sympathetic nervous system control and local release of noradrenaline, leading to vasoconstriction [37], [38]. Our tissue pO_2_ measurements lend additional new evidence of the kidney's distinct behavior: tissue oxygen increased only for hyperoxia but not during hypercapnia as seen in the brain and liver. When elevated CO_2_ is inhaled, because the expected vasodilatory response found in many other organs is absent, tissue oxygenation also does not increase. Lack of response in both perfusion and tissue pO_2_ manifests as negligible changes in T_1_ and T_2_*. When elevated O_2_ is inhaled, arterial blood becomes saturated with oxygen, there is a drastic increase in tissue pO_2_, and the venous Hb/HbO_2_ drops. These changes give rise to a T_1_ decrease and T_2_* increase, which are responses consistent in organs with an arterial blood supply.

The effects of breathing carbogen are much more difficult to predict, and many conflicting reports exist in the literature even within the same organ. The reason for the complexity is that although both O_2_ and CO_2_ are used to enhance oxygen supply, the vasodilatory or vasoconstrictive effects of combined O_2_ and CO_2_ are not easily predicted. Although the CO_2_ component should theoretically counteract any vasoconstrictive effects of oxygen and maintain adequate blood flow, blood flow can be substantially lower compared to using CO_2_ alone, as seen in the liver in this study. Even a small perfusion reduction can abolish the advantage of using a hyperoxic gas, a fact often overlooked in MRI studies. In the future, the independent effects of O_2_ and CO_2_ in a carbogen mixture can be investigated in greater detail by grading the gas components. For the purpose of this study, the use of carbogen helped to demonstrate the independent and organ-specific effects of O_2_ and CO_2_.

It should be noted that invasive measurements can rapidly probe the dynamics of physiological response not achievable with other methods, including imaging. Also, although invasive probes cannot faithfully capture heterogeneity of tissue response due to limited tissue sampling, they remain the most practical means to validate organ response even if only on an overall level. These invasive measurements can then establish the baseline interpretation of MRI measurements so that additional information from imaging on response heterogeneity can be properly evaluated. In this study, a single probe was used per organ to maintain similar experimental conditions for measuring both liver and kidney response. The consistent results obtained across animals suggest that overall organ responses likely dominated over any heterogeneity within the organ. It should also be noted that while our OxyFlo perfusion measurements can be used to infer changes in blood volume (since perfusion changes are mediated through vasodilatation or vasoconstriction), we did not directly measure blood volume in this study. Another important note is that a simultaneous study involving invasive probes and MRI is difficult to perform in this setting, because although the surgical procedures can be performed on the animal outside the MRI scanner, many of the surveillance devices used for monitoring the animal's physiological status could not be brought into the scan room. Furthermore, the tools used to fix the position of the probes relative to tissue were not all MRI-compatible and the setup required more space than was available within the magnet bore. Beyond these logistical issues, there is also the problem of imaging an open abdomen when the tissue regions being probed are in close proximity to the interface between the organ and air. These challenges may well explain why there is limited information in the literature on simultaneous MRI and physiological monitoring. In this study, we seek to fill this gap by performing invasive experiments under experimental conditions similar to those in our previous MRI study [11].

In addition to conventional MRI assessment using changes in T_1_ and T_2_* relaxation times, ^19^F MRI [39] is an emerging technique for direct measurement of tissue oxygen. It can potentially overcome the challenge of separating out changes in blood flow and vascular volume from the measurement of oxygen content. Measurement of increased oxygenation in multiple organs in response to hyperoxia was recently demonstrated using this technique [40]. The authors noted increases in tissue oxygen that were higher compared to our study; this difference may be due to different species or to the use of mechanical ventilation.

The findings of this study are most likely translatable to humans. Aside from a significantly higher heart rate, the rabbit physiology is similar to human's in terms of blood supply. For example, both species have a dual hepatic input. Since an organ's arterial and venous supplies are the primary conduits to manipulate gas challenge responses, the results seen in the rabbit liver and kidney in this study should be representative of those in humans. A potential source of variation from a human setting is the use of anesthesia in animals. Anesthetic drugs may have an impact on vascular response. For this reason, isoflurane was chosen over other alternatives (e.g. pentobarbital, which is vasoconstrictive) to ensure minimum disturbance to the resting perfusion state.

In conclusion, this study provided new evidence on organ-specific tissue response to hyperoxia and hypercapnia in the liver and kidney. Perfusion measurements were consistent with known physiological behavior of these organs, as CO_2_-mediated vasodilatation was present in the liver but not in the kidney. Dynamic tissue oxygenation measurements revealed response characteristics that are consistent with the unique MRI measurements reported in these organs. Liver oxygenation responded to elevated CO_2_ but not elevated O_2_. This can be attributed to the buffering action of the portal vein. Therefore, blood flow, not hyperoxia, is the primary influence of liver oxygenation. In contrast, kidney oxygenation responded to elevated O_2_ but not elevated CO_2_. This can be attributed to the absence of flow-mediated oxygen delivery. Therefore, hyperoxia, not blood flow, is the primary influence of kidney oxygenation. With improved insight into the unique physiological response in the liver and kidney, we have a better understanding of the association between MRI and different organ response mechanisms, ultimately extending the use of MRI relaxation times as robust non-invasive biomarkers in various organs.
